# Supplementary material for: Diversity and evolution of cerebellar folding in mammals
Source: eLife. 2023 Sep 22;12:e85907. doi: 10.7554/eLife.85907 (PMC10617990; doi:10.7554/eLife.85907)
Supplement: Supplementary file 2. [file elife-85907-supp2.pdf]

Partial correlations

K. Heuer, N. Traut, A. de Sousa, S. Valk, J. Clavel, R. Toro, June 2023

Partial correlations are used to measure the strength of a relationship between two variables while controlling for the effect of one or more other variables. In other words, partial correlation is the correlation between two variables after removing the effect of one or more independent factors on both variables.

In our manuscript, we used partial correlations to better understand the correlation structure of our variables. This notebook is divided in 2 parts, the first one provides an illustration of how partial correlations can be computed, and what the meaning of negative partial correlations may be. The second part aims at evaluating the statistical power we had to detect partial correlations as significant as those we observed (a posteriori power analysis).

1. The computation of partial correlations

The partial correlation coefficient can be calculated as follows:

`r_ab.c = (r_ab - r_ac.r_bc) / sqrt((1 - r_ac^2).(1 - r_bc^2))`

where:

- `r_ab.c` is the partial correlation coefficient between `a` and `b`,

controlling for `c`.

- `r_ab` is the correlation coefficient between `a` and `b`.
- `r_ac` is the correlation coefficient between `a` and `c`.
- `r_bc` is the correlation coefficient between `b` and `c`.

Even if the correlation between `a` and `b` is strong and positive, their partial correlation could be 0 or even negative. Consider, for example, the case where the 3 vectors of measurements `a`, `b`, `c` resulted from the combination of uncorrelated random vectors `x`, `y`, `z`. Suppose that `a = 0.5 x + 0.2 y + 0.1 z`, `b = 0.5 x - 0.2 y + 0.1 z`, and `c = x`. The measurements a and b will be positively correlated because of the effect of `x` and `z`. However, if we compute the residuals of a and b after covarying the effect of `c` (i.e., `x`), their partial correlation will be negative because of the opposite effect of `y` on `a` and `b`.

The code that follow demonstrate this example.

For more information on partial correlations see https://en.wikipedia.org/wiki/Partial_correlation

In [ ]:
```python
import numpy as np
import matplotlib.pyplot as plt
import statsmodels.api as sm
```

In [ ]:
```python
x = np.random.randn(50)
y = np.random.randn(50)
z = np.random.randn(50)

a = 0.5*x + 0.2*y + 0.1*z
b = 0.5*x - 0.2*y + 0.1*z
X = np.vstack((a, b, x)).T
```

Here we compute the partial correlations following the definition from the introduction, computing the residuals, and then computing their correlation. The partial correlation is negative, even though their correlation is positive:

In [ ]:
```python
# fit a ~ x, b ~ x
ax = sm.OLS(a, x).fit().resid
bx = sm.OLS(b, x).fit().resid
print(f"correlation a~b: {np.corrcoef(a, b)[0,1]}")
print(f"partial correlation a~b|x: {np.corrcoef(ax, bx)[0,1]}")
```

```
correlation a~b: 0.501779198289467
partial correlation a~b|x: -0.8110773754985613
```

Here we use an alternative method for computing partial correlations exhaustively among many variables. This is the method that is actually used in our paper. The results for the 3 variables in the example is the same:

In [ ]:
```python
def partialcorr(X):
    C = np.cov(X, rowvar=False, bias=True)
    C_inv = np.linalg.inv(C)
    d = np.sqrt(np.diag(C_inv))
    P = -C_inv / np.outer(d, d)
    np.fill_diagonal(P, 1)
    return P
```

In [ ]:
```python
partialcorr(X)
```

Out[ ]:
```
array([[ 1.        , -0.81207564,  0.92710747],
       [-0.81207564,  1.        ,  0.94327373],
       [ 0.92710747,  0.94327373,  1.        ]])
```

Finally, the plots show graphically that the correlation between `a` and `b` are positive, but their partial correlation is negative:

In [ ]:
```python
plt.figure(figsize=(15, 5))
plt.subplot(131); plt.plot(x, a, 'o'); plt.title('a ~ x')
plt.subplot(132); plt.plot(x, b, 'o'); plt.title('b ~ x')
plt.subplot(133); plt.plot(a, b, 'o'); plt.title('a ~ b')

plt.figure(figsize=(5, 5))
plt.plot(ax, bx, 'o'); plt.title('a ~ b | x')
```

Out[ ]:
```
Text(0.5, 1.0, 'a ~ b | x')
```

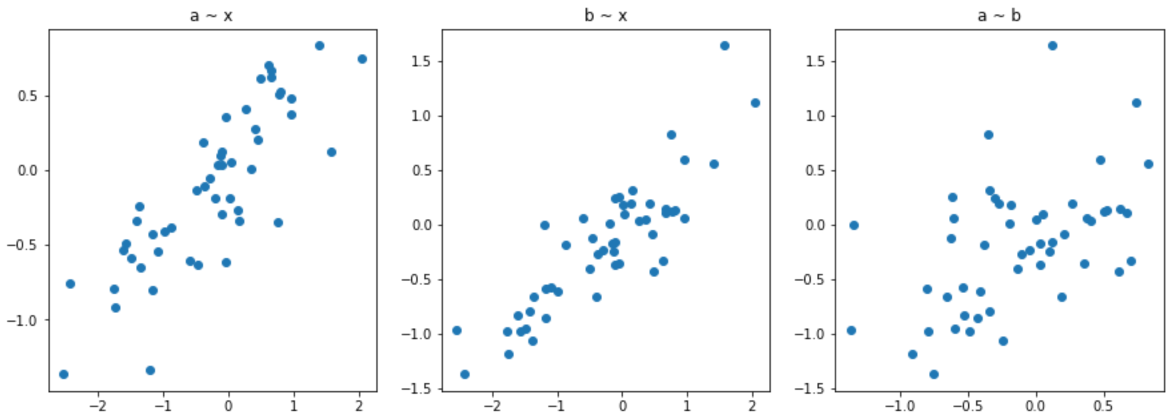

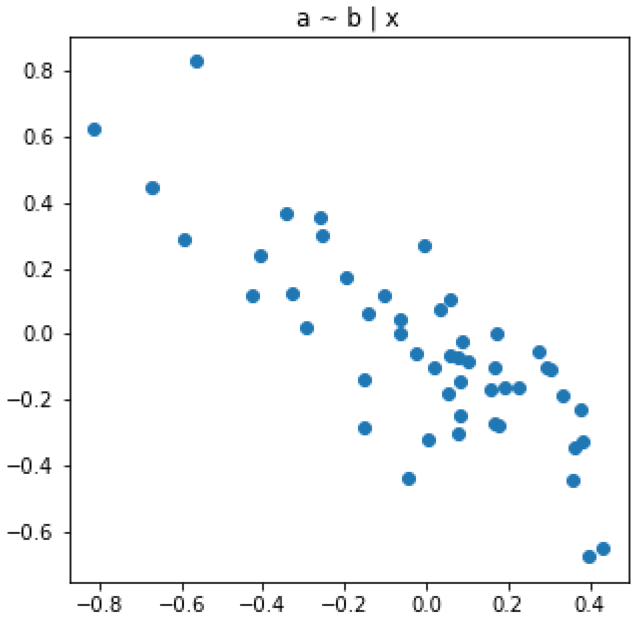

2. Statistical power

We used 9 different variables to study neuroanatomical diversity in our sample of 56 species. These variables are not independent, on the contrary, they vary following a very strong pattern. This pattern captures their multivariate allometric scaling and is represented by the 1st principal component of the variance-covariance matrix of the 9 variables. As we saw previously, the inversion of this matrix leads to the simultaneous estimation of all partial correlations among our variables. We estimated the significance of these partial correlations using the edge exclusion test introduced by Whittaker (1990).

Here we use simulations to evaluate the statistical power we had to detect partial correlations as large as those we observed.

In [ ]:
```
# This is the variance-covariance matrix of our variables, which can also

C = np.array([
       [5.68999487, 3.75035486, 1.05249198, 0.78099597, 1.07278117, 0.763
       [3.75035486, 2.78186834, 0.7534478 , 0.57310966, 0.79035063, 0.570
       [1.05249198, 0.7534478 , 0.22899229, 0.17110868, 0.22659353, 0.157
       [0.78099597, 0.57310966, 0.17110868, 0.13481407, 0.172311,   0.121
       [1.07278117, 0.79035063, 0.22659353, 0.172311  , 0.24049524, 0.170
       [0.76312705, 0.57093027, 0.15711043, 0.12163194, 0.17068113, 0.129
```

```
        [0.1646759 , 0.11573696, 0.03739531, 0.02511677, 0.03669998, 0.022
        [0.12895582, 0.09413106, 0.02805883, 0.01699588, 0.02990122, 0.017
        [0.23629373, 0.16357597, 0.04728047, 0.03265633, 0.04789162, 0.032
    ])
```

In [ ]:

```
# cov2pcor: covariance to partial correlations
def cov2pcor(C):
    C_inv = np.linalg.inv(C)
    d = np.sqrt(np.diag(C_inv))
    P = -C_inv / np.outer(d, d)
    np.fill_diagonal(P, 1)
    return P
```

In [ ]:

```
# edge_exclusion_test
from scipy.stats import chi2

def edge_exclusion_test(C, n):
    m = cov2pcor(cov2cor(C))
    mt = m[np.triu_indices(len(m), 1)]
    edges = -n * np.log(1 - mt**2)
    res = chi2.sf(edges, df=1)
    resu = np.around(res, decimals=4)
    # print(resu.shape)

    rows, cols = (len(m), len(m))
    M = [[0 for i in range(cols)] for j in range(rows)]
    for ind, (row, col) in enumerate(zip(*np.triu_indices(len(m), 1))):
        M[row][col] = resu[ind]
        M[col][row] = "***" if resu[ind] <= 0.001 else "**" if resu[ind]
    return M
```

The following function computes our power (a.k.a. sensitivity) to detect the partial correlations implied by the variance-covariance matrix `Ctarget`, for a given strength `effect_start`, and a given alpha (significance) threshold `min_stars`. The function scans sample sizes from 20 to up to 100 species, running each time 1000 simulations. For every simulation we generate data using the variance-covariance matrix Ctarget with the different sample sizes. Significance is estimated using the edge exclusion test. We keep track of the number of true positives (where the significance in of one partial correlation in the simulated data matches that of the observed data), false negatives, false positives and true negatives. The function returns a vector of sensitivity versus sample size, and specificity versus sample size (another value that's useful for evaluating the behaviour of a test).

In [ ]:

```
n_stars = lambda s: s.count('*')

def power_for_effect_size(Ctarget, effect_stars=3, min_stars=1):
    sensitivity, specificity = [], []
    for n_samples in range(20, 105, 5):
        TP, FP, TN, FN = 0, 0, 0, 0
        for _ in range(1000):
            D = np.random.multivariate_normal(np.zeros(9), Ctarget, n_sam
            C2 = np.cov(D)
            P2 = cov2pcor(C2)
            res2 = edge_exclusion_test(C2, n_samples)
            for s, s2 in zip(sum(res, []), sum(res2, [])):
```

```
                if type(s) != str:
                    continue
                if n_stars(s) >= effect_stars:
                    if n_stars(s2) >= min_stars:
                        TP += 1
                    else:
                        FN += 1
                if n_stars(s) < effect_stars:
                    if n_stars(s2) >= min_stars:
                        FP += 1
                    else:
                        TN += 1
        sensitivity.append(TP/(TP+FN)*100)
        specificity.append(TN/(FP+TN)*100)
    return sensitivity, specificity
```

In [ ]:

```
# pca2cov: pca to covariance matrix
def pca2cov(vecs, vals):
    return np.dot(vecs, np.dot(np.diag(vals), vecs.T))
```

In [ ]:

```
# power to detect partial correlations in the observed data
power_large, _ = power_for_effect_size(C, 3, 1)
power_medium, _ = power_for_effect_size(C, 2, 1)
power_small, _ = power_for_effect_size(C, 1, 1)

# create a variance-covariance matrix with weakened allometry
test_vals = vals.copy()
test_vals = vals**0.5
g = np.prod(vals)/np.prod(test_vals)
test_vals = test_vals*g**(1/len(test_vals))
C1 = pca2cov(vecs, test_vals)
P1 = cov2pcor(C1)

# power to detect partial correlations in the data with weakened allometr
m_power_large, _ = power_for_effect_size(C1, 3, 1)
m_power_medium, _ = power_for_effect_size(C1, 2, 1)
m_power_small, _ = power_for_effect_size(C1, 1, 1)
```

In [ ]:

```
plt.figure(figsize=(16, 6))

x = np.arange(20, 105, 5)

plt.subplot(121)
plt.plot(x, power_large, 'o-', label='large effect size')
plt.plot(x, power_medium, 'o-', label='medium effect size')
plt.plot(x, power_small, 'o-', label='small effect size')
plt.xlabel('Sample size')
plt.ylabel(f'Power')
plt.xlim(15, 105)
plt.ylim(30, 105)
plt.grid("on")
plt.legend(loc=4, bbox_to_anchor=(0.95, 0.05))
plt.tight_layout()
plt.plot([56, 56], [30, 105], 'k--')
plt.text(54, 60, 'N=56', horizontalalignment='center', verticalalignment=
plt.title('a. Power to detect the observed effect size')

plt.subplot(122)
plt.plot(x, m_power_large, 'o-', label='large effect size')
```

```
plt.plot(x, m_power_medium, 'o-', label='medium effect size')
plt.plot(x, m_power_small, 'o-', label='small effect size')
plt.xlabel('Sample size')
plt.ylabel(f'Power')
plt.xlim(15, 105)
plt.ylim(30, 105)
plt.grid("on")
plt.legend(loc=4, bbox_to_anchor=(0.95, 0.05))
# tight display
plt.tight_layout()
plt.plot([56, 56], [30, 105], 'k--')
plt.text(54, 75, 'N=56', horizontalalignment='center', verticalalignment=
plt.title('b. Power to detect data with weaker allometry')

# plt.savefig('../data/derived/supp.fig.power.svg', dpi=300)
```

Out[ ]: Text(0.5, 1.0, 'b. Power to detect data with weaker allometry')

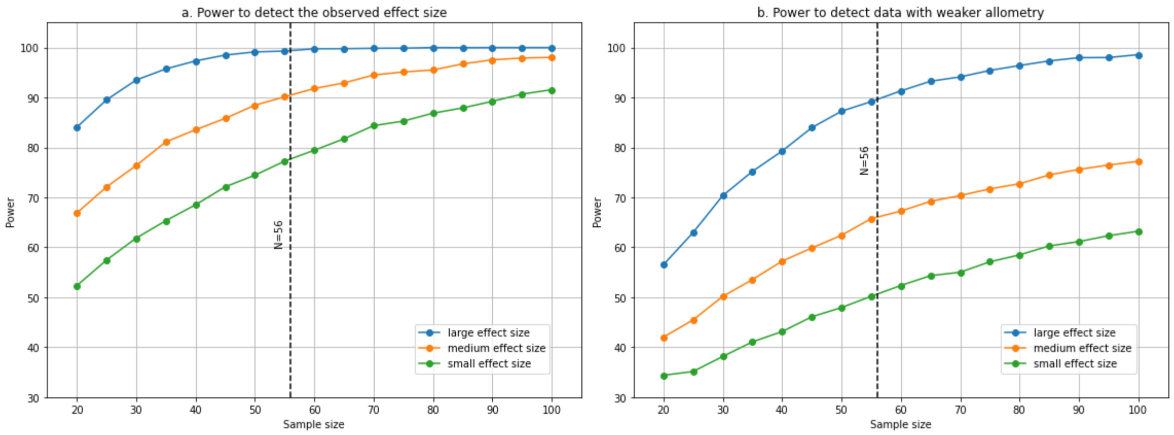

In [ ]:
