# Supplementary figures and images for: Diversity and evolution of cerebellar folding in mammals
Source: eLife. 2023 Sep 22;12:e85907. doi: 10.7554/eLife.85907 (PMC10617990; doi:10.7554/eLife.85907)

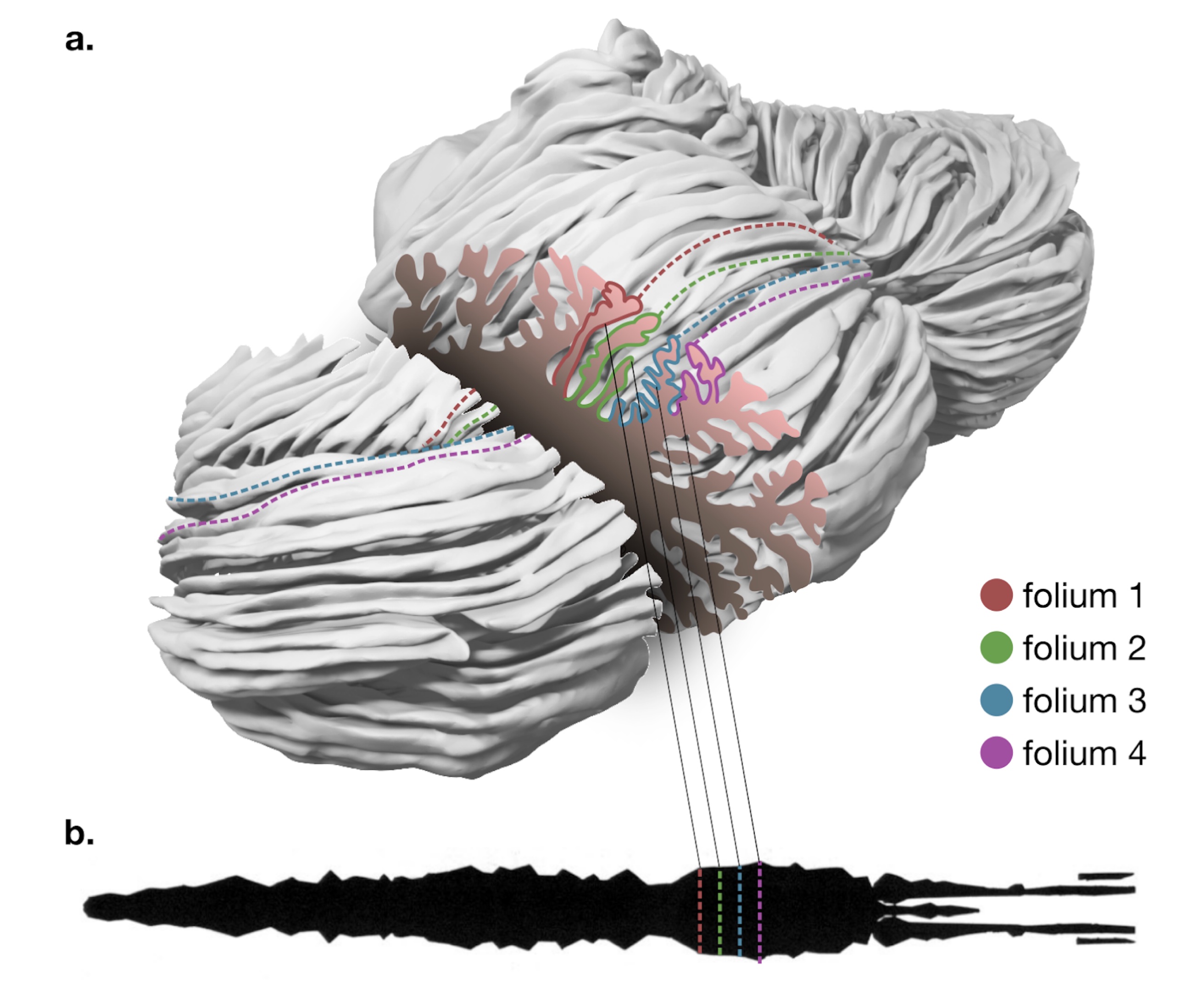

Supplement: Supplementary file 3. [file elife-85907-supp3.jpg]
